# Effects of Dietary Energy Sources on *Post Mortem* Glycolysis, Meat Quality and Muscle Fibre Type Transformation of Finishing Pigs

**DOI:** 10.1371/journal.pone.0131958

**Published:** 2015-06-30

**Authors:** Yanjiao Li, Jiaolong Li, Lin Zhang, Changning Yu, Meng Lin, Feng Gao, Guanghong Zhou, Yu Zhang, Yuanfang Fan, Lina Nuldnali

**Affiliations:** 1 College of Animal Science and Technology, Nanjing Agricultural University, Nanjing, Jiangsu, 210095, China; 2 Key Laboratory of Animal Origin Food Production and Safety Guarantee of Jiangsu Province, Nanjing Agricultural University, Nanjing, Jiangsu, 210095, China; 3 Synergetic Innovation Center of Food Safety and Nutrition, Nanjing Agricultural University, Nanjing, Jiangsu, 210095, China; University of Lleida, SPAIN

## Abstract

Dietary energy source can influence muscle glycogen storage at slaughter. However, few studies have demonstrated whether the diet-induced change of muscle glycogen is achieved by the transformation of muscle fibre type. This study investigated the effects of dietary energy sources on meat quality, *post mortem* glycolysis and muscle fibre type transformation of finishing pigs. Seventy-two barrows with an average body weight of 65.0 ± 2.0 kg were selected and were allotted to three iso-energetic and iso-nitrogenous diets A, B or C, and each treatment consisted of three replicates (pens) of eight pigs each. Diet A contained 44.1% starch, 5.9% crude fat and 12.6% neutral detergent fiber (NDF); diet B contained 37.6% starch, 9.5% crude fat and 15.4% NDF; and diet C contained 30.9% starch, 14.3% crude fat and 17.8% NDF. The duration of the experiment was 28 days. After feed withdrawal 12 h, 24 pigs (eight per treatment) were slaughtered, samples from M. longissimus lumborum (LL) were collected for subsequent analysis. The results showed that pigs fed diet C had lesser average daily gain, average daily feed intake and back fat depth than those fed diet A (*P*<0.05). Diet C increased pH_45min_ (*P*<0.05) and decreased drip loss (*P*<0.05) in LL muscles compared with diet A. Meat from pigs fed diet A showed increased contents of lactate and greater glycolytic potential (GP) compared with those fed diet C (*P*<0.05). Greater mRNA expression of myosin heavy-chain (MyHC)-I and IIa and lesser expression of MyHC-IIx and IIb (*P*<0.05) in LL muscles were found in pigs fed diet C, than in pigs fed diet A. In addition, pigs fed diet C resulted in downregulation of miR23a and upregulation of miR409 and miR208b (*P*<0.05), associated with conserved changes of their corresponding targets. These findings indicated that diets containing low starch and high fibre were beneficial in reducing muscle glycolysis, improving meat quality of finishing pigs. This reduction of GP may be partially associated with the improvement of oxidative fibre composition in LL muscle, and the change in myofibre type may be correlated with the change in the miRNA expression.

## Introduction

Feeding strategy is an effective means of improving pork quality. Numerous studies have shown that different dietary energy sources could influence muscle glycogen storage at slaughter [1,2], which further influences meat quality through affecting the rate and extent of *post mortem* pH decline. Despite external feed manipulation, meat quality is largely dependent on internal myofibre types [3] because the inherent differences of mATPase activity, glycolytic enzyme profiles and glycogen contents exist in different muscle fibre types [4]. In general, skeletal muscles are categorized into four fibre types, based on myosin heavy-chain (MyHC) ATPase: MyHC-I (slow-oxidative), MyHC-IIa (fast-oxidative), MyHC-IIx (intermediary to MyHC-IIa and MyHC-IIb) and MyHC-IIb (fast-glycolytic) [5]. Indeed, the increased proportion of MyHC-IIb fibre in pork resulted in rapid *post mortem* pH decline, greater lightness and drip loss [6]. Additionally, muscle fibre type composition could also be affected by diets. Men et al., [7] reported that dietary supplementary with conjugated linoleic acid increased MyHC-I level in muscle of finishing pigs. Moreover, microRNAs (miRNAs) are certified to be involved in the regulation of muscle fibre types [8]. MiRNAs are ~22 nucleotides long non-coding RNAs and inhibit mRNA translation or promote mRNA degradation by bounding to the 3’-untranslated regions (UTRs) of target mRNAs [9]. Until now, little literature has been available on whether the diet-induced change of muscle glycogen is achieved by the transformation of muscle fibre type and whether this transformation is associated with the change in the miRNA expression. Thus, this study was designed to determine meat quality, *post mortem* glycolysis and muscle fibre type transformation in pigs feeding different energy source of finishing diets.

## Materials and Methods

### Animal Treatments and Experimental Diets

A total of 72 barrow (Duroc×Landrace×Yorkshire, DLY) pigs with similar initial body weight (average BW = 65.0 ± 2.0 kg) were selected from the Swine Research Institute (Changxing branch) of Nanjing Agricultural University. All pigs were uniformly allotted into three iso-energetic and iso-nitrogenous experimental diet treatments, with three replicates per treatment and eight pigs per replicate. All diets were formulated to meet the nutrient requirements of National Research Council (NRC 2012) [10] for finishing pigs. The traditional diet (diet A) of finishing pigs was used as the control diet, the starch content of which was 44.1%. The starch contents of the diets B and C were 37.6% and 30.9%, respectively. The starch content was formulated by decreasing approximately 15% or 30%, on the basis of diet A. In the case of iso-energetic diets, the contents of dietary fat and neutral detergent fiber (NDF) varied with the change of dietary starch content. Diet A contained 44.1% starch, 5.9% crude fat and 12.6% NDF; diet B contained 37.6% starch, 9.5% crude fat and 15.4% NDF; diet C contained 30.9% starch, 14.3% crude fat and 17.8% NDF. The nutrient composition was calculated according to the recommendations of “Chinese Feed Database” [11] and analysed using AOAC procedures [12]. The nutrient levels of the experimental diets were shown in Table 1. Diets were fed in mash form and feed and water were available *ad libitum*. All pigs were housed in solid concrete floor pens (3.40 m × 4.80 m) in a temperature-controlled room. The experimental design and procedures were approved by the Animal Care and Use Committee of Nanjing Agricultural University.

**Table 1:** The composition and nutrient content of the experimental diets provided for finishing pigs (%).

Item	Diet		
	A	B	C
**Ingredients**			
**Maize**	69.0	46.1	26.5
**Wheat bran**	5.2	10.1	7.3
**Soybean meal**	14.0	11.4	9.5
**Rice bran**	8.8	27.1	50.0
**Soybean oil**	0.6	2.9	4.3
**Lysine-HCL**	0.02	—	—
**Limestone**	0.78	0.93	1.00
**Dicalcium phosphate**	0.3	0.2	0.2
**Salt**	0.3	0.3	0.3
**Premix** ^**†**^	1.0	1.0	1.0
***Calculated nutrient level***			
**Net energy (MJ/kg)**	10.3	10.3	10.4
**Lys**	0.74	0.74	0.77
**Met**	0.22	0.23	0.24
**Trp**	0.16	0.17	0.16
**Thr**	0.52	0.51	0.52
**Calcium**	0.52	0.52	0.52
**Available phosphorus**	0.18	0.18	0.20
***Analysed nutrient level***			
**Dry matter**	85.4	85.3	86.6
**Crude protein**	14.3	14.2	14.2
**Starch**	44.1	37.6	30.9
**Crude fat**	5.9	9.5	14.3
**NDF**	12.6	15.4	17.8
**Ash**	4.6	6.0	7.6

^†^The premix provided per kilogram of diet: 100 mg of iron as iron sulphate, 100 mg of zinc as zinc oxide, 30 mg of manganese as manganous oxide, 20 mg of copper as copper sulphate, 0.3 mg of selenium as sodium selenite, 0.5 mg of iodine as calcium iodate, 1720 μg retinyl acetate, 25 μg cholecalciferol, 8.0 mg DL-α-tocopheryl acetate, 3.0 mg menadione sodium bisulphite, 2.0 mg thiamin mononitrate, 6.0 mg riboflavin, 3.0 mg pyridoxine hydrochloride, 30 mg nicotinic acid, 30 mg calcium pantothenate, 1.0 mg folic acid, 20 μg cyanocobalamin, 300 mg choline.

### Growth Performance and Carcass Traits

Initial and final body weights and feed consumption per pen were recorded on days 0 and 28 of the experiment to calculate average daily weight gain (ADG), average daily feed intake (ADFI) and feed conversion ratio (FCR). ADG = (total final body weights per pen − total initial body weights per pen) / 8 pigs / 28 days, ADFI = total feed intake per pen / 8 pigs / 28 days, FCR = ADFI / ADG. On the day of slaughter, after a 12-h fast, 24 pigs (eight per treatment, two or three per pen) were randomly selected, weighed and simultaneously transported (approximately 30 min) to a slaughterhouse. Pigs were electrically stunned, exsanguinated, scalded, depilated, labelled, eviscerated and cut down the midline accurately. The hot carcass weight (Not including the head, feet, tail and offal, but remaining the leaf fat and kidney) of individual pigs was directly recorded on the assembly line, to calculate dressing percentage (= hot carcass weight / live weight × 100%) before the carcasses were sent to the chilling room (4°C). In the chilling room, back fat depth was measured opposite the first rib, lumbar and last rib, and their average values were taken as the back fat thickness.

### Sample Collection

At 45 min *post mortem*, samples of M. longissimus lumborum (LL) taken at the last rib of the left carcass were immediately frozen in liquid nitrogen for the analysis of glycolytic potential (GP) and the expressions of RNA and miRNA. Thereafter, the LL muscles from the 8th to 12th ribs of the left carcass were removed, trimmed of subcutaneous fat and connective tissue and vacuum packed at 4°C for the measurement of meat quality.

### Glycolytic Potential

The determination of glycogen was conducted as described by Zhang et al., [13]. Briefly, a frozen muscle sample (0.5 g) was ice bath homogenized in 4.5 mL ice-cold perchloric acid solution (0.85 M HClO_4_) at 13,500 rpm for 30 s and then centrifuged (2,700 g, 4°C, 10 min). The supernatant was neutralized with 10 M KOH, and then the glycogen in the supernatant fraction was hydrolysed to glucose, by incubation with amyloglucosidase and the glucose-6-phosphate was catalysed to glucose, by incubation with glucose-6-phosphatase. Finally, the glucose was measured with a commercial glucose oxidase kit (Shanghai RongSheng Biotech Co. Ltd., Shanghai, China). The lactate content in the muscle was measured using a commercial diagnostic kit (Nanjing Jiancheng Bioengineering Institute, Nanjing, China), in accordance with the manufacturer’s instructions. The glycolytic potential (GP) was calculated according to the formula from Monin and Sellier [14] as follows: GP = 2 × glycogen + lactate.

### Meat Quality Measurements

The muscle pH at 45 min (pH_45min_) and 24 h (ultimate pH, pHu) *post mortem* were measured, using a pH meter (HI9125 portable waterproof pH/ORP, HANNA Instruments, Cluj-Napoca, Romania). The meat colour was measured at 24 h *post mortem* from a cut surface using a chromameter CR-400 (Konica Minolta, Sensing Inc., Osaka, Japan). The chromameter was set to D65 illuminant using a 0° viewing angle and an 8-mm diameter viewing area. Mean Commission Internationale de L’Eclairage (CIE) L* (lightness), a* (redness) and b* (yellowness) values were collected from three different locations on the cut surface of each sample. The drip loss was determined at 24 h *post mortem*. Briefly, the long length of a muscle section was cut along the fibre direction (2 cm × 3 cm × 5 cm), trimmed of fat, weighed, suspended in a polyethylene plastic bag, ensuring that the sample did not make contact with the bag and then stored at 4°C. After 24 h, the sample was reweighed to calculate drip loss percentage, as described by Honikel [15].

After measuring drip loss, at 48 h *post mortem*, the meat samples were again weighed and placed in plastic bags and cooked in a 75°C water bath (Julabo77960, labortechnik, Gmb1-1, Germany) to reach an internal temperature of 70°C. The internal temperature of the meat was monitored using a thermocouple during the cooking process. Then, the meat was cooled in running water to room temperature, wiped with absorbent paper to remove residual moisture and weighed to calculate cook loss percentage, as described by Honikel [15]. Then, the cooked samples were immediately cut parallel to the muscle fibre direction into shaped strips (1 cm × 1 cm × 3 cm) for shear force measurement using a shear apparatus (C-LM3B, Engineering College of Northeast Agricultural University, Harbin, China) with a load cell of 15 kg and a crosshead speed of 200 mm min^-1^. Six replicates of each sample were measured to calculate the average value.

### Bioinformatics Analysis

We used TargetScan (http://www.targetscan.org) and PicTar (http://pictar.mdc-berlin.de/) to predict the target genes of miR499, 208b and 23a. As pig (sus. sucrofa) genes are not included in the current versions of TargetScan and PicTar, the predictions were based on the human mRNA/miRNA interactions. Human myocyte enhancer factor 2C (MEF2C) and peroxisome proliferator-activated receptor γ coactivator 1α (PGC-1α) were predicted to be the targets of miR23a, specificity protein 3 (Sp3) and thyroid hormone receptor associated protein 1 (Thrap1) were predicted to be the targets of miR208b, SRY-box containing gene 6 (Sox6) was predicted to be the target of miR409. The 3’UTR sequences of target genes and the primer sequences of miRNAs were shown in Table 2. It can be seen that the seed region conservations of miRNAs in pig were as same as human. By homology comparison (http://blast.ncbi.nlm.nih.gov/Blast.cgi), the pig Sox6, Sp3, PGC-1α, MEF2C and Thrap1 cDNA sequences were shown to have 95%, 97%, 94%, 95% and 95% identity, respectively, with the human versions.

**Table 2:** The primer sequences of miRNAs and the 3’UTR sequences of target genes.

Item	miRNA or mRNA	Sequences
**miRNA**	hsa-mir-23a-3p	65-CCUUUAGGGACCG**UUACACU**A-45
	ssc-mir-23a	62-CCUUUAGGGACCG**UUACACU**A-42
**miR23a target genes**	PGC-1α 3’UTR	3491-AGCCAUGUACUAU**AAUGUGA**U-3511
	MEF2C 3’UTR	1932-UUCCUUCUCUUGG**AAUGUGA**A-1952
**miRNA**	hsa-mir-208b-3p	67-UGUUUGGAAAACAA**GCAGAAU**A-46
	ssc-mir-208b	66-UGUUUGGAAAACAA**GCAGAAU**A-45
**miR208b target genes**	Sp3	811-AAUAAGGUGUAUUG**CGUCUUA**G-831
	Thrap1	551-AAAAUAUAUGUAAU**CGUCUUA**A-572
**miRNA**	hsa-mir-499a-5p	53-UUUGUAGUGACGU**UCAGAAU**U-33
	ssc-mir-499-5p	35-UUUGUAGUGACGU**UCAGAAU**U-15
**miR499 target genes**	Sox6	1198-GAUAUAGGUAUAU**AGUCUUA**C-1218

*PGC-1α* peroxisome proliferator-activated receptor γ coactivator 1α, *MEF2C* myocyte enhancer factor 2C, *Sp3* specificity protein 3, *Thrap1* thyroid hormone receptor associated protein 1, *Sox6* SRY-box containing gene 6.

### Total RNA Isolation

Total RNA was isolated from frozen LL muscle samples using Trizol reagent (Takara Biotechnology Co. Ltd., Dalian, China) according to the manufacturer’s protocol. The purity and quantity of total RNA were measured by a NanoDrop 1000 spectrophotometer (Thermo Scientific, Wilmington, DE, USA) at 260 and 280 nm.

### mRNA Expression Analyses

Total RNA was treated with DNase I (Takara Biotechnology Co. Ltd.) to remove DNA and reverse transcribed to cDNA (10 μL reaction system for maximum use of 500 ng of Total RNA) using a PrimeScript RT Master Mix kit (Takara Biotechnology Co. Ltd.), according to the manufacturer’s instructions. The reactions were incubated for 15 min at 37°C, followed by 5 s at 85°C to inactivate the RT enzyme. Real-time PCR was carried out in optical 96-well plates on an ABI 7500 Real-Time PCR System (Applied Biosystems, Foster City, CA) using SYBR Premix Ex Taq Kits (Takara Biotechnology Co. Ltd.). Primers used for real-time PCR are presented in Table 3 and were synthesized by Invitrogen. The amplification was performed in a total volume of 20 μL, containing 10 μL of SYBR Premix Ex Taq, 0.4 μL of each primer (10 μM), 0.4 μL of ROX Reference Dye II, 2 μL of cDNA and 6.8 μL of sterilized doubled-distilled water. The program was as follows: 95°C for 30 s, followed by 40 cycles of 95°C for 5 s and 60°C for 34 s and the collection of the fluorescence signal at 60°C. The amplification of 18s rRNA was used for each sample to normalize the expression of the selected genes. After amplification, melt curve analysis was performed to validate the specificity of the reactions. Relative gene expression was calculated using the 2^−ΔΔCt^ method, as described by Livak and Schmittgen [16].

**Table 3:** Sequences used for real-time PCR primers.

Gene	Primer sequence (5' to 3')	Product size (bp)	GenBank accession no.
**MyHC- I**	Forward: CGTGGACTACAACATCATAGGC		
	Reversed: CTTTGCCCTTCTCAACAGGT	152	NM_213855
**MyHC- IIa**	Forward: AAACCTCACGGAAGAGATGG		
	Reversed: TCAGGGTGTTGACTTTGTCCT	134	NM_214136
**MyHC- IIx**	Forward: GAAACCGTCAAGGGTCTACG		
	Reversed: CGCTTCCTCAGCTTGTCTCT	153	NM_001104951
**MyHC- IIb**	Forward: GATGTTCCTGTGGATGGTCA		
	Reversed: CTCGTTGGTGAAGTTGATGC	148	NM_001123141
**Sox6**	Forward: GCAGCAAGGGTCAAGAGTG		
	Reversed: GGCAGTTCAGGGTAAAGGTG	130	XM_003122960
**Sp3**	Forward: CAGATGGTCAGCAGGTTCAG		
	Reversed: TAGCAGGAGGTGTTCCAGAG	133	XM_001928783.5
**PGC-1α**	Forward: AGGGAAGAATACCGCAGAGA		
	Reversed: TGTCCGTGTTGTGTCAGGTC	135	NM_213963
**MEF2C**	Forward: CGCTCTTCATCTTGGGTCAG		
	Reversed: CGTGTGTTGTGGGTATCTCG	121	NM_001044540.1
**Thrap1**	Forward: CATCCCTGAAGCACACAGTC		
	Reversed: AACATCGGCACCCTTGATA	130	XM_005669005
**18s rRNA**	Forward: GAGACGGTGGGACAGCG		
	Reversed: GCCCTCGGTCGAGTTGTC	162	AY265350

*MyHC* myosin heavy chain, *Sox6* SRY-box containing gene 6, *Sp3* specificity protein 3, *PGC-1α* peroxisome proliferator-activated receptor γ coactivator 1α, *MEF2C* myocyte enhancer factor 2C, *Thrap1* thyroid hormone receptor associated protein 1.

### miRNA Expression Analyses

MiRNAs were quantified using a miRCURY LNAUniversal RT microRNA PCR starter kit (Exiqon, Vedbaek, Denmark) according to the manufacturer’s protocol. Briefly, 10 ng of total RNA was reverse transcribed in 10 μL reactions and incubate for 60 min at 42°C, followed by 5 min at 95°C to inactivate the RT enzyme. For quality control of the cDNA synthesis, UniSp6 spike-in was added to each RT reaction and later quantified using control primers included in the kit. Primers for miR409, miR208b and miR23a were using LNA PCR primer sets by Exiqon. The primer sequences for hsa-miR-499a-5p, hsa-miR-208b-3p and hsa-miR-23a-3p were shown in Table 3. Real-time PCR was performed using ABI 7500 Real-Time PCR System (Applied Biosystems) and the conditions recommended by Exiqon. The program was as follows: 95°C for 10 min, followed by 40 cycles of 95°C for 10 s and 60°C for 1 min. The amplification of UniSp6 (miRCURY LNA Universal RT microRNA PCR starter kit comes with) was used for each sample to normalize the expression of the selected miRNAs. After amplification, melt curve analysis was performed to validate the specificity of the reactions. Relative gene expression was calculated using the 2^−ΔΔCt^ method, as described by Livak and Schmittgen [16].

### Statistical Analyses

Data analysis was performed by ANOVA with the SAS statistical software (version 9.2 for Windows, Institute Inc., Cary, NC). The growth performance data were analysed with the pen as the experimental unit, and the carcass traits, meat quality, lactic acid, glycogen, GP and expressions of MyHC isoform genes, miRNAs and their target genes data were analysed with the individual pig as the experimental unit (n = 8). The significant difference among treatments was compared by Tukey test. The results were presented as the mean values and the SEM. All statements of significance are based on a *P*-value less than 0.05.

## Results

### Growth Performance and Carcass Traits

As shown in Table 4, pigs fed diet A had higher ADFI and ADG (*P*<0.05) than pigs fed diet C, whereas no significant difference in FCR was observed among the three treatments (*P*>0.05). Pigs fed diet C had lower back fat depths compared with those fed diet A (*P*<0.05). However, dietary energy sources did not affect carcass weights and carcass yields of finishing pigs.

**Table 4:** Effect of dietary energy sources on the growth performance and carcass traits of finishing pigs.

Item	Diet^†^				
	A	B	C	SEM	P-value
**ADG (kg/d)**	0.80^a^	0.77^ab^	0.73^b^	0.01	0.031
**ADFI (kg/d)**	2.77^a^	2.51^ab^	2.40^b^	0.07	0.042
**FCR (kg/kg)**	3.49	3.27	3.30	0.08	0.548
**Final live weight (kg)**	92.0	91.8	90.2	0.8	0.616
**Carcass weight (kg)**	67.7	67.0	65.9	0.8	0.686
**Carcass yield (%)**	73.6	72.9	73.1	0.3	0.636
**Back fat (mm)**	19.5^a^	19.1^ab^	17.5^b^	0.3	0.020

^a,b^ Means within a row with no common superscript differ significantly (*P*<0.05).

^†^Diet A contained 44.1% starch, 5.9% crude fat and 12.6% NDF; diet B contained 37.6% starch, 9.5% crude fat and 15.4% NDF and diet C contained 30.9% starch, 14.3% crude fat and 17.8% NDF.

FCR = feed conversion ratio (feed: gain, kg:kg).

ADG, ADFI and FCR were analysed with pen as the experimental unit (n = 3);

Final live weight, Carcass weight, Carcass yield and Back fat were analysed with the individual pig as the experimental unit (n = 8).

### Glycolytic Potential

In this study, compared with the diet A, diet C decreased the content of lactate and GP (*P*<0.05), but did not affect the glycogen content in LL muscles (Table 5).

**Table 5:** Effect of dietary energy sources on muscle glycolytic potential of finishing pigs at 45 min postmortem.

Item (μmol/g)	Diet^†^				
	A	B	C	SEM	P-value
**Lactate**	74.6^a^	71.7^ab^	68.4^b^	1.0	0.038
**Glycogen**	14.5	14.5	14.0	0.5	0.938
**GP**	103.5^a^	100.6^ab^	96.4^b^	1.2	0.045

^a,b^ Means within a row with no common superscript differ significantly (*P*<0.05).

^†^Diet A contained 44.1% starch, 5.9% crude fat and 12.6% NDF; diet B contained 37.6% starch, 9.5% crude fat and 15.4% NDF and diet C contained 30.9% starch, 14.3% crude fat and 17.8% NDF.

GP (glycolytic potential) = 2 × glycogen + lactate (Monin and Sellier, 1985).

GP, lactate and glycogen are on a fresh-tissue basis.

### Meat Quality

Compared with the diet A, pigs fed diet C showed an increase in values of pH_45min_ in LL muscle (*P*<0.05) (Table 6). In addition, diet C resulted in a decrease in drip loss of LL muscles (*P*<0.05), compared with diet A. There were no significant differences of pHu, meat color, cooking losses and shear force of LL muscles in all of treatments.

**Table 6:** Effect of dietary energy sources on M. longissimus lumborum meat quality of finishing pigs.

Item	Diet^†^				
	A	B	C	SEM	P-value
**pH**_**45min**_	6.29^b^	6.40^ab^	6.42^a^	0.03	0.041
**pH**_**24h**_	5.59	5.58	5.60	0.02	0.833
Lightness (L*)	43.4	43.3	43.2	0.3	0.968
Redness (a*)	5.60	5.78	5.87	0.10	0.584
Yellowness (b*)	1.66	1.65	1.87	0.06	0.288
**Drip loss (%)**	2.05^a^	1.95^ab^	1.85^b^	0.03	0.028
**Cooking loss (%)**	27.3	27.0	26.9	0.5	0.947
**Shear force (N)**	34.4	36.6	37.1	1.6	0.796

^a,b^ Means within a row with no common superscript differ significantly (*P*<0.05).

^†^Diet A contained 44.1% starch, 5.9% crude fat and 12.6% NDF; diet B contained 37.6% starch, 9.5% crude fat and 15.4% NDF and diet C contained 30.9% starch, 14.3% crude fat and 17.8% NDF.

All traits in this table were analysed with the individual pig as the experimental unit (n = 8).

### MyHC Isoform Genes Expressions

**Fig 1:**
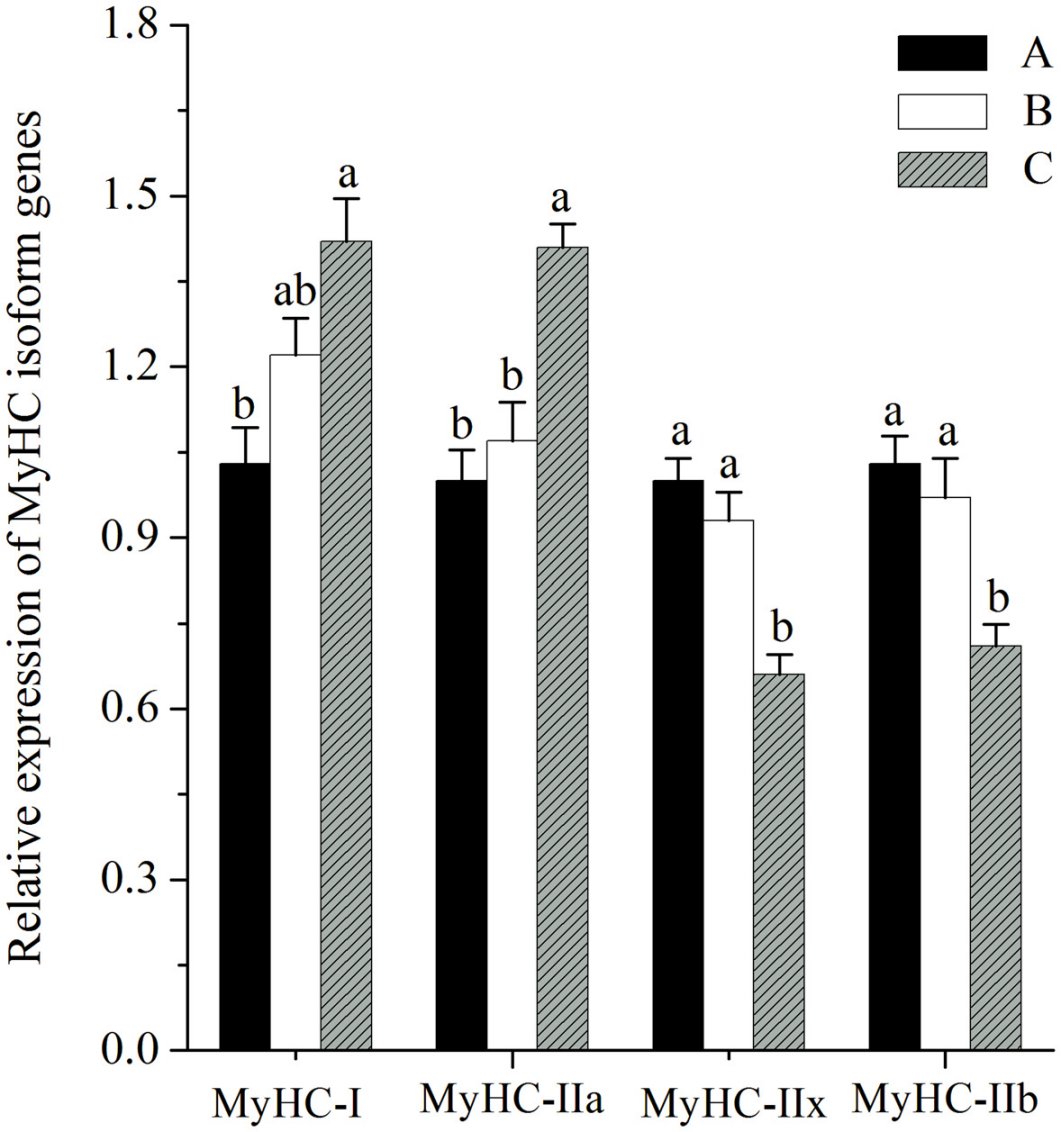
Relative mRNA expression of myosin heavy-chain (MyHC) isoform genes in LL muscles. Diet A contained 44.1% starch, diet B contained 37.6% starch and diet C contained 30.9% starch. mRNA expression was normalized to 18s rRNA gene expression. Data were shown as the mean ± standard error of eight replicates. ^a,b^Mean values within different letters were significantly different (*P*<0.05).

As shown in Fig 1, the mRNA expression of MyHC-I was greater in pigs fed diet C, than in pigs fed diet A (*P*<0.05). The mRNA expression of MyHC-IIa was higher in pigs fed diet C compared with pigs fed diets A and B (*P*<0.05). The mRNA expressions of MyHC-IIx and MyHC-IIb were lower in pigs fed diet C compared with pigs fed diets A and B (*P*<0.05).

### miRNAs and Their Corresponding Target Gens Expressions

**Fig 2:**
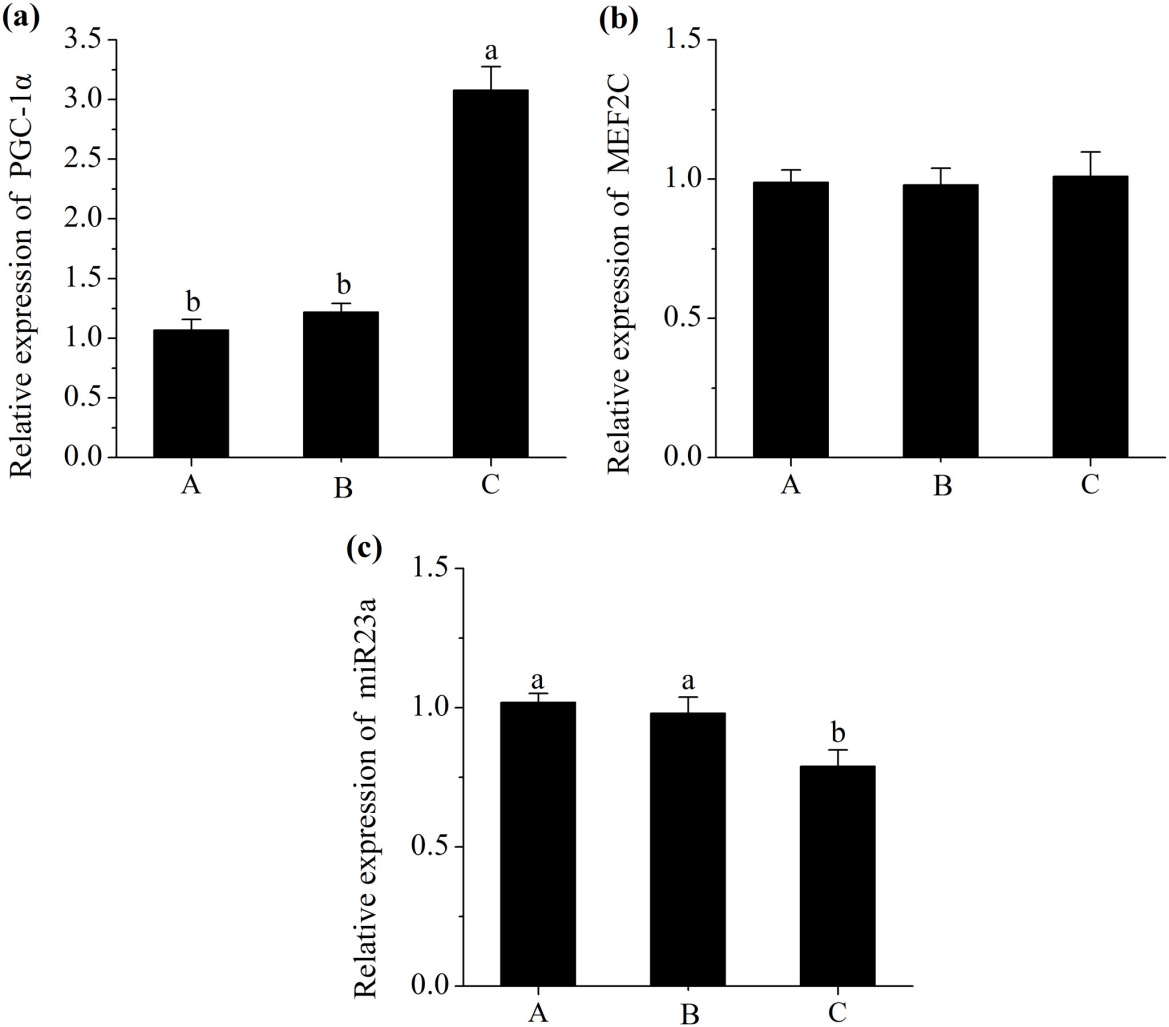
Relative mRNA expression of proliferator-activated receptor γ coactivator 1α (PGC-1α) (a), Relative mRNA expression of myocyte enhancer factor 2C (MEF2C) (b) and Relative miRNA expression of miR23a (c). Diet A contained 44.1% starch, diet B contained 37.6% starch and diet C contained 30.9% starch. mRNA expressions were normalized to 18s rRNA gene expression. miRNA expression was normalized to UniSp6 expression. Data were shown as the mean ± standard error of eight replicates. ^a,b^Mean values within different letters were significantly different (*P*<0.05).

**Fig 3:**
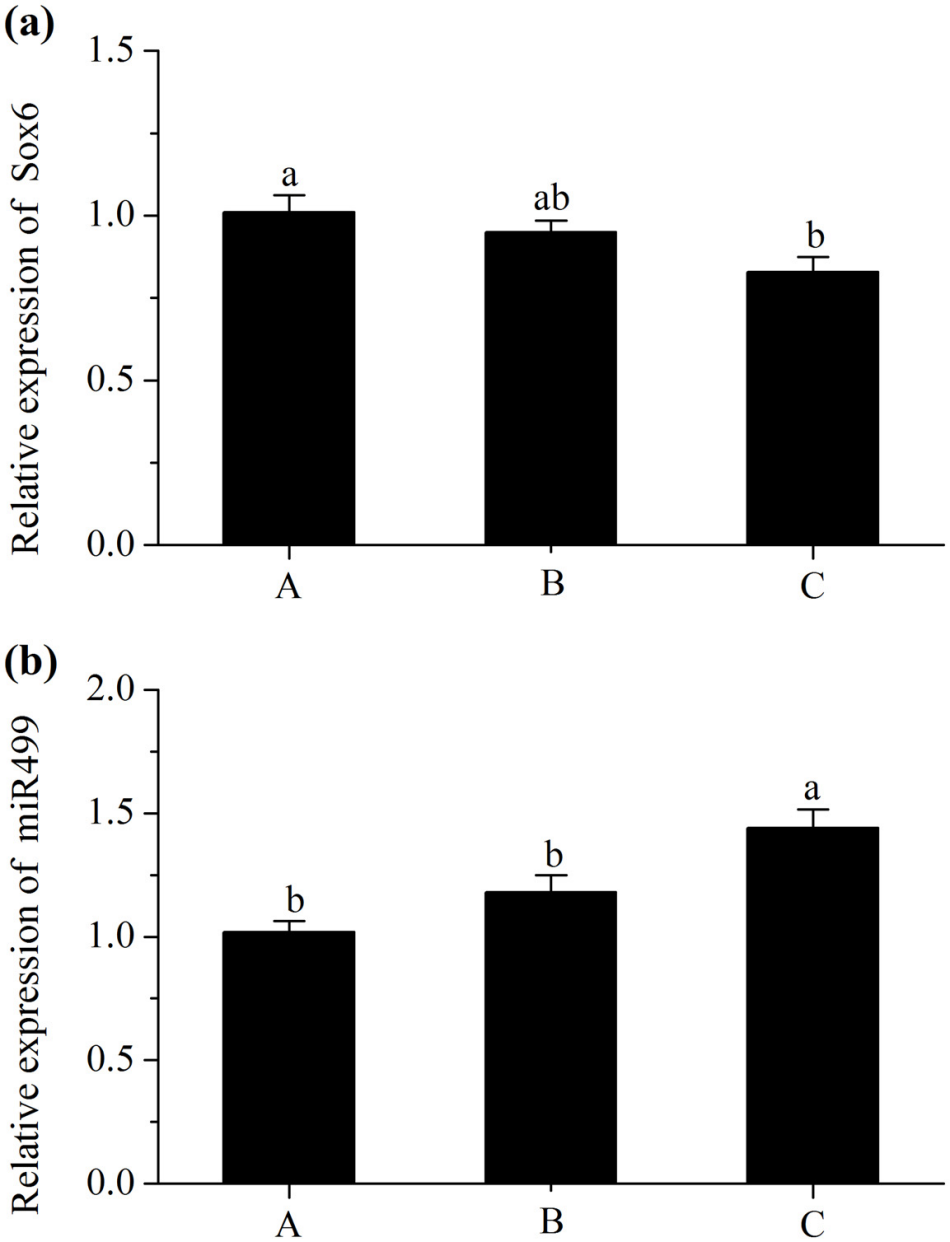
Relative mRNA expression of SRY-box containing gene 6 (Sox6) (a) and Relative miRNA expression of miR499 (b). Diet A contained 44.1% starch, diet B contained 37.6% starch and diet C contained 30.9% starch. mRNA expression was normalized to 18s rRNA gene expression. miRNA expressions were normalized to UniSp6 expression. Data were shown as the mean ± standard error of eight replicates. ^a,b^Mean values within different letters were significantly different (*P*<0.05).

**Fig 4:**
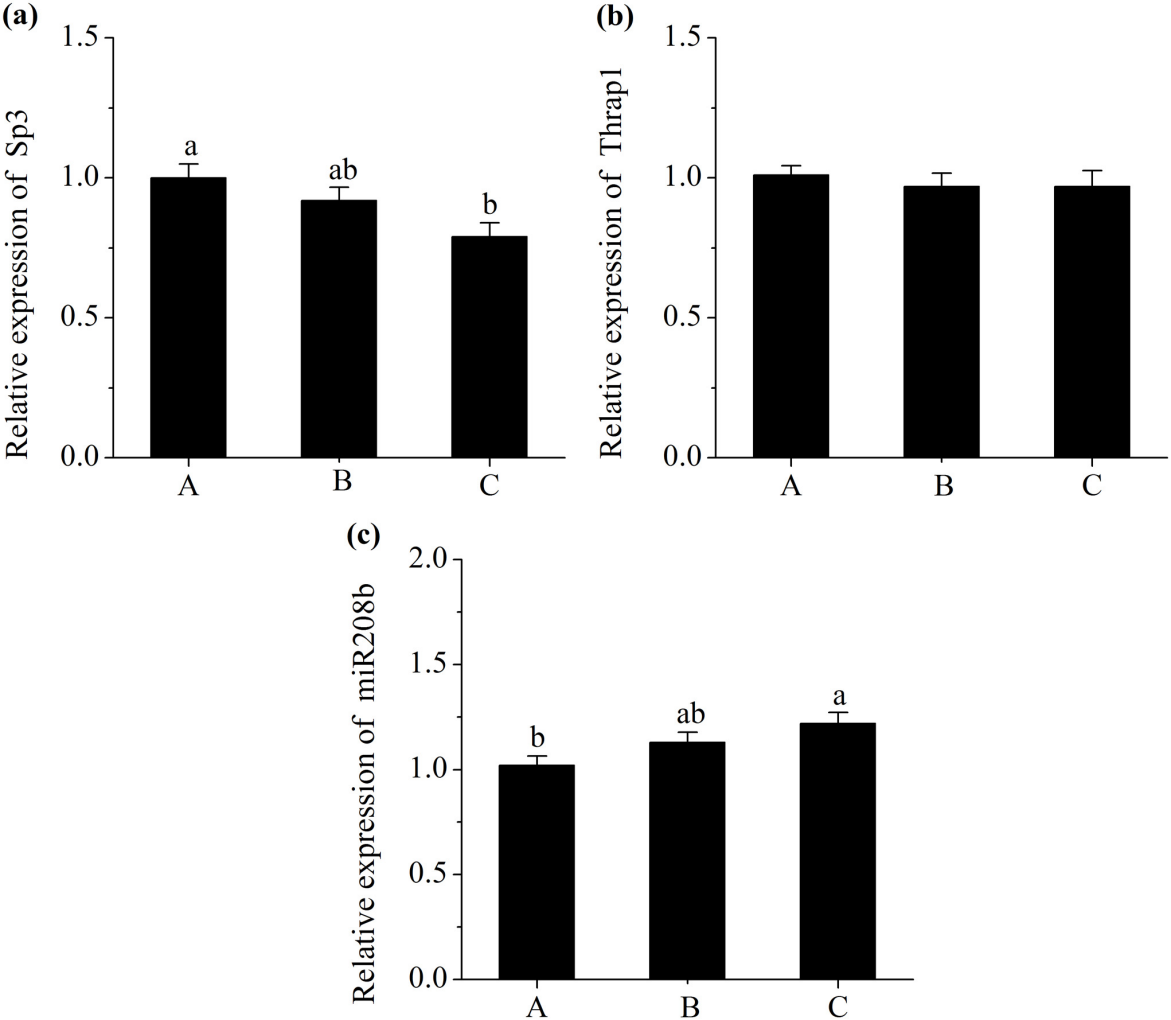
Relative mRNA expression of specificity protein 3 (Sp3) (a), Relative mRNA expression of thyroid hormone receptor associated protein 1 (Thrap1) (b) and Relative miRNA expression of miR208b (c). Diet A contained 44.1% starch, diet B contained 37.6% starch and diet C contained 30.9% starch. mRNA expression was normalized to 18s rRNA gene expression. miRNA expressions were normalized to UniSp6 expression. Data were shown as the mean ± standard error of eight replicates. ^a,b^Mean values within different letters were significantly different (*P*<0.05).

A decrease in the expression of miR23a (*P*<0.05; Fig 2c) and an increase in the level of PGC-1α (*P*<0.05; Fig 2a) were observed in pigs fed with diet C. However, the level of MEF2C did not differ in treatments (*P*>0.05; Fig 2b). Additionally, pigs fed diet C had a higher abundance of miR499 compared with those fed diet A (*P*<0.05; Fig 3b), and had a lower mRNA expression of Sox6 (*P*<0.05; Fig 3a). Moreover, pigs fed diet C had a greater abundance of miR208b (*P*<0.05; Fig 4c), and had a lesser mRNA expression of Sp3 (*P*<0.05; Fig 4a). However, No significant difference in expression of Thrap1 (*P*>0.05; Fig 4b) was found in treatments.

## Discussion

This study clearly showed that pigs fed the high fibre and fat diet exhibited reduced ADFI than pigs fed the high starch diet. The reduction of ADFI in the present study may be attributed to the poor palatability [1] and early satiety [17] induced by the high fibre diet. Similarly, Bertol et al., [18] also reported that pigs fed high fibre diets had less feed intake compared with those fed corn and soybean-based diets. Although the higher soybean oil in diet can improve feed efficiency [19] and decrease heat increment [20], in this study, diet C did not prevent the reduction of ADG, nor was it beneficial to FCR. The inaction of diet treatments on FCR might be due to the antagonistic effects of dietary fibre content and fat levels on feed efficiency. Moreover, the main determinant of growth rate in pigs is daily energy intake [21], and in this study, diets were formulated to meet iso-proteic and iso-energetic standards, thus the decrease in ADG could be ascribed to less feed intake. In addition, the present study showed that back fat thickness of pigs was also decreased by diet C, which was likely associated with decreased lipogenic rates in pigs fed the high fat, high fibre diets [22]. This reduction of lipogenic rates may be attributed to the decrease in proportions of first synthetized fatty acid (palmitate and oleate) induced by lower dietary carbohydrate, and the decrease in the activities of key lipogenic enzymes induced by higher dietary fiber [22].

Muscle glycogen stores at the time of slaughter and the rate of *post mortem* glycolysis affect the rate and extent of *post mortem* pH decline which further influences meat quality [2]. In the present study, GP together with lactate concentration of LL muscle at 45 min *post mortem* were decreased by the higher fibre and fat diet, whereas there were no changes in corresponding glycogen contents. These data were contrary to the results of Rosenvold et al., [1], who reported that pigs fed diets with lower level of digestible carbohydrate (compared with a standard diet) reduced muscle glycogen contents. Additionally, the insulin resistance induced by high fat feeding can reduce glucose uptake and glycogen synthesis in muscle [18]. Thus, the muscle glycogen content should not be unchanged by the low starch high fat diet in the current study. For this, one possible explanation might be that glycogen contents were higher, but more rapidly degraded, in pigs fed a high starch diet during the early stages of *post mortem* muscle metabolism, resulting in similar glycogen contents, but higher lactate contents at 45 min *post mortem*. Furthermore, Ylä-Ajos et al., [23] reported that the activities of the glycogen debranching enzyme and glycogen phosphorylase (key enzymes of *post mortem* glycogenolysis) were increased within the higher fast muscle fibre type, so the greater proportion of fast muscle fiber observed in pigs fed diet A in the present study (Fig 1) indicated that feeding such a diet enhanced glycogenolysis rate at the early stages of *post mortem*.

In this study, pigs fed low starch and high fibre and fat diet led to greater pH_45min_, without affecting the pH_u_ or the meat color in LL muscle, which was in agreement with the studies by Rosenvold et al., [1,24]. These results indicated that the rapid glycolysis rate at the early stage of *post mortem* resulted in lower pH_45min_ but not in pH_u_. In addition, Scheffler et al., [25] also showed that GP was weakly associated with pH_u_. In the present study, the meat of pigs fed high fibre and fat diet had a lower drip loss than those fed high starch diet. This observation was consistent with the study of Rosenvold et al., [24], who reported that drip loss was closely related to early *post mortem* pH.

It is well known that muscle fibre types have an important impact on meat quality. Although the total number of skeletal muscle fibres is fixed in animals before birth [26], the composition of muscle fibre types varies along the animal’s life to adapt to different physiological requirements [27]. Actually, muscle fibre type transformation is a complex process with many muscle structure and metabolic genes involved in [28], and data published recently have indicated that miRNAs also play a key role in the transformation [29]. To our knowledge, this is the first study to investigate the relationship between miRNA expression and myofibre transformation in finishing pigs, following the different dietary energy source treatment.

In the current study, pigs fed diet C, with low starch and high fat showed greater proportion of oxidative fibre types (MyHC-I, IIa) and lesser composition of glycolytic fibre types (MyHC-IIx, IIb) (Fig 1). As shown in the studies of Turner et al., [30] and de Wilde et al., [31], high-fat diets could promote oxidative capacity and fast-to-slow fibre type transformation in skeletal muscle via the PGC-1α related signalling pathway. PGC-1α is known to be involved in mitochondrial biogenesis, oxidative metabolism and fast-to-slow skeletal muscle fibre transformation [28]. Moreover, Hanke et al., [32] reported that the reduction of glucose supply in muscle cell culture could increase the PGC-1α expression and promote myotubes metabolism trend to oxidative metabolism. Additionally, Hanke et al., [33] found that the high glucose in skeletal muscle cell culture could result in a partial slow-to-fast fibre transformation. In this study, the decreased concentration of plasma glucose (data not shown) in pigs fed diet C might play some role in stimulating changes in muscle fibre activity and eventually in muscle fibre type composition. In addition to the direct regulation of PGC-1α by dietary energy metabolite (fatty acid, glucose), PGC-1α was also shown to be a target gene of miR23a [34]. Furthermore, Safdar et al., [34] reported that miR23a expression was downregulated, paralleled with the upregulated expression of PGC-1α in skeletal muscle of mice experiencing endurance exercise. Additionally, MEF2C, which is also a target gene of miR23a, has been shown to be a promotion of slower muscle phenotype [35]. However, in the present study, no significant difference in expression of MEF2C was found in treatments. Overall, the decreased level of miR23a concurrent with the increased level of PGC-1α in LL muscles of pigs fed diets C may be associated with the increased slow fibre type composition.

Despite the elevation of slow fibre type proportion achieved by increased PGC-1α expression, there are some transcriptional repressors to inhibit slow fibre gene expression including Sox6, Sp3 and Thrap1. Fortunately, Sox6 can be inhibited by miR499, Sp3 and Thrap1 can be inhibited by miR208b [36,37]. MiR208b and miR499 are encoded by introns of their host myosin genes, Myh7 and Myh7b, respectively. Some previous studied reported that during myogenesis of Sox6 mutant mice, the expressions of slow myofibre genes were activated whereas fast myofibre genes were repressed [38,39]. In addition, it has been proposed that the decreased expression of slow MyHC induced by Sp3 was achieved by the binding of Sp3 proteins to slow MyHC proximal promoter [40]. Furthermore, the Thrap1, which functions as a thyroid hormone receptor cofactor, appears to be repressed by miR208 and subsequently activate the slow fiber specific gene expression [37]. Based on these studies, it was concluded that the repressions of Sox6 by miR499, or Sp3 and Thrap1 by miR208b may be associated with the increase in the expressions of slow MyHC genes. In the present study, miR499 and miR208b showed greater expressions in pigs fed low starch and high fat diets, concurrent with decreased levels of Sox6 and Sp3, whereas the level of Thrap1 was not differ in treatments. These results demonstrated that the post-transcriptional degradation of target genes by miR499 or miR208b may be associated with the fast-to-slow fibre transformation.

In conclusion, finishing pigs fed iso-energetic and iso-nitrogenous diets containing low starch and high dietary fibre and fat could decrease their growth performance, back fat depth and drip loss of LL muscles as well as the muscle GP. This elevation in meat quality and reduction in glycolysis capacity may be partially correlated with the transformation of fast-to-slow fibre in LL muscle, and this transformation may be associated with the change in the miRNA expression.

## Supporting Information

S1 Table The minimal data for growth performance of finishing pigs. (XLSX)

S2 Table The minimal data for carcass traits of finishing pigs. (XLSX)

S3 Table The minimal data for muscle glycolytic potential of finishing pigs. (XLSX)

S4 Table The minimal data for meat quality of finishing pigs. (XLSX)

S5 Table The minimal data for mRNA expressions of MyHC isoform genes. (XLSX)

S6 Table The minimal data for expressions of miR23a, PGC-1α and MEF2C. (XLSX)

S7 Table The minimal data for expressions of miR499 and Sox6. (XLSX)

S8 Table The minimal data for expressions of miR208b, Sp3 and Thrap1. (XLSX)
